# A novel composition of two heterozygous *GFM1* mutations in a Chinese child with epilepsy and mental retardation

**DOI:** 10.1002/brb3.1791

**Published:** 2020-08-09

**Authors:** Cuiping You, Na Xu, Shiyan Qiu, Yufen Li, Liyun Xu, Xia Li, Li Yang

**Affiliations:** ^1^ Central Laboratory Linyi People's Hospital Linyi China; ^2^ Department of Pediatrics Linyi People's Hospital Linyi China

**Keywords:** epilepsy, *GFM1*, *GFM1*‐linked disease, mitochondrial translation

## Abstract

**Introduction:**

G elongation factor mitochondrial 1 (*GFM1*) encodes one of the mitochondrial translation elongation factors. *GFM1* variants were reported to be associated with neurological diseases and liver diseases in a few cases. Here, we present a novel composition of two heterozygous mutations of *GFM1* in a boy with epilepsy, mental retardation, and other unusual phenotypes.

**Methods:**

The patient was found to be blind and experienced recurrent convulsive seizures such as nodding and hugging at the age of 3 months. After antiepileptic treatment with topiramate, he had no obvious seizures but still had mental retardation. The patient vomited frequently at 16 months old, sometimes accompanied by epileptic seizures. Hematuria metabolic screening, mutation screening of mitochondrial gene, and mitochondrial nuclear gene were negative. Then, he was analyzed by whole‐exome sequencing (WES).

**Results:**

Whole‐exome sequencing revealed a novel composition of two heterozygous mutations in *GFM1*, the maternal c.679G > A (has not been reported) and the paternal c.1765‐1_1765‐2del (previously reported). At present, there is no specific and effective treatment for the disease, and the prognosis is very poor.

**Conclusion:**

The discovery of new phenotypes and new genotypes will further enrich the diagnosis information of the disease and provide more experiences for clinicians to quickly diagnose the disease and judge the prognosis.

## Graphical abstract

We present a novel compound heterozygous mutation of GFM1 with c.679G > A and c.1765‐2_1765‐1delAG deletion in a boy with epilepsy, mental retardation, and other symptoms. We found GFM1‐linked disease can involve multiple systems and has a variety of clinical manifestations. The relationship between GFM1 gene mutation and clinical phenotype, as well as the effective treatment methods, need to be further studied. Our report expands the mutation and clinical spectrum of GFM1.

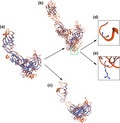

## 1 INTRODUCTION

Mitochondria are the energy factories of eukaryotic cells, which contain thousands of proteins that maintain their specific functions. These proteins are encoded by mitochondrial DNA and the nuclear genome. Pathogenic mutations in these mitochondrial genes can cause multiple serious diseases (Scheffer et al., 2017; Thompson et al., 2020). Mitochondrial DNA encodes its own mRNA, rRNA, and tRNA, to synthesize some of the proteins it needs. Proteins encoded by mitochondrial DNA are involved in the composition of oxidative phosphorylation system complexes, so any gene mutation that affects the replication, transcription, and translation of mitochondrial DNA may cause oxidative phosphorylation deficiency (de Laat, Rodenburg, & Smeitink, 2014). Mitochondrial translation is crucial for maintaining mitochondrial function. Besides tRNAs and rRNAs encoded by mitochondria, the translation of mitochondrial DNA requires many initiation, extension, and termination factors. For instance, the initiation factors *MTIF2* (mitochondrial translational initiation factor 2) and *MTIF3* (mitochondrial translational initiation factor 3), and the extension factors *TUFM* (Tu translation elongation factor)*, TSFM* (mitochondrial elongation factor Ts), *GFM1,* and *GFM2* (G elongation factor mitochondrial 2), are components of the mitochondrial translation system (Kuzmenko et al., 2014). Among these genes, the variants of elongation factors *TUFM*, *TSFM, GFM1*, and *GFM2* were reported to be associated with mitochondrial diseases, mainly causing oxidative phosphorylation deficiency diseases. Mutations of *TUFM* had been reported to be associated with combined oxidative phosphorylation deficiency resulting in lactic acidosis, fatal encephalopathy, and cardiomyopathy (Hershkovitz et al., 2019; Valente et al., 2007). Mutations in *TSFM* were associated with combined oxidative phosphorylation deficiency 3 with symptoms including seizures, ataxia, and tremor (Ahola et al., 2014; Scala et al., 2019). *GFM1* mutations were associated with combined oxidative phosphorylation deficiency 1 (Barcia et al., 2020). Diseases associated with *GFM2* included combined oxidative phosphorylation deficiency 39 and mitochondrial metabolism disease (Fukumura et al., 2015; Glasgow et al., 2017).

The nuclear gene *GFM1*, located at 3q25.32, consists of 18 exons and encodes protein mtEFG1 (elongation factor G, mitochondrial). As one of the mitochondrial elongation factors, *GFM1* catalyzes the translocation of peptidyl‐tRNAs from the ribosomal A site to the P site during the elongation phase of mitochondrial translation (Barcia et al., 2020; Gao et al., 2001). There have been many reports that *GFM1* mutations are associated with combined oxidative phosphorylation deficiency disease (Calvo et al., 2012; Simon et al., 2017; Smits et al., 2011). The combined oxidative phosphorylation deficiency caused by *GFM1* gene mutation involves multiple systems (brain, liver, eyes, etc.) and has various clinical manifestations (seizure, hepatomegaly, mental retardation, etc.). At present, there is no specific and effective treatment for the disease, and the prognosis is very poor. The relationship between *GFM1* gene mutation and clinical phenotype, as well as the effective treatment methods, needs to be further studied. In this case report, we first describe a child with a novel composition of two heterozygous mutations of *GFM1* gene, c.679G > A at exon 5 and c.1765‐2_1765‐1delAG deletion at exon 15 (NM_024996). With the two mutations, the patient exhibited symptoms of epilepsy, mental retardation, lactic acidosis, and other unusual phenotypes. In this paper, we will present the case in detail. Detailed analysis of phenotypes and the new genotype will further enrich our understanding of *GFM1*‐linked disease.

## 2 CASE PRESENTATION

### 2.1 Subjects

The patient was a 3‐year‐old boy, who suffered from recurrent seizures for 2 years and 8 months and suffered from recurrent vomiting for 1 year and 7 months. At the age of 3 months, he was found to be blind. At 3 months and 20 days old, he showed a series of seizures during his awakening period. These seizures consisted of nodding, clasping, and squinting to the right, with 2–3 strings a day, 7–8 times a string, and 7–8 strings a day at most. VEEG (video electroencephalogram) result showed highly dysrhythmic brain waves and caught two tonic–clonic seizures. Brain MRI showed no obvious abnormality. Since then, he received antiepileptic treatment. Sodium valproate was ineffective, treatment was switched to topiramate, and the dosage was gradually increased. At 6 months old, he had no obvious seizures but still had mental retardation.

At the age of 1 year and four months, he began to vomit frequently, accompanied by adduction flexion of both upper limbs, trembling of limbs, gaze, and cyanosis around the mouth, which lasted for about half an hour. Because of recurrent vomiting accompanied by focal seizures, he was frequently admitted to the hospital, and each hospitalization examination showed obvious blood acidosis. Urine screening showed hyperlactic acidemia with increased ketonuria, glycosuria, and several organic acids. Blood screening showed that Ala, Phe/Tyr, CO, C2, C3, and C4‐OH increased and Gly, Leu/Ile, Trp, Thr, Val, Gly/Ala, and Val/Phe decreased. After that, the patient developed heavy breathing symptoms, exhibited during sleep. Electrolyte levels were checked in the hospital, and CO_2_ was found to be 12.3 mmol/L. After the symptomatic treatment of energy, levocarnitine, ambroxol, and so on, he was discharged after improvement. After discharge, the child still had frequent epileptic seizures for a period of time and continued to take topiramate 25 mg q12h orally. Hematuria metabolic screening, mutation screening of mitochondrial gene, and mitochondrial nuclear gene were negative. The respiratory chain enzyme activity of the exceptional blood was normal. At the age of one year and four months, VEEG showed abnormal EEG: highly irregular electroencephalogram, slow background activity, and paroxysmal δ activity in bilateral frontal and occipital regions during sleep. Brain MRI showed an abnormal signal in the left pontine arm, thickening of the cerebral cortex, decrease in the medulla, enlargement of the ventricles, widening of the sulci and fissures, and increase in DWI signal in subcortical white matter.

**FIGURE 1:**
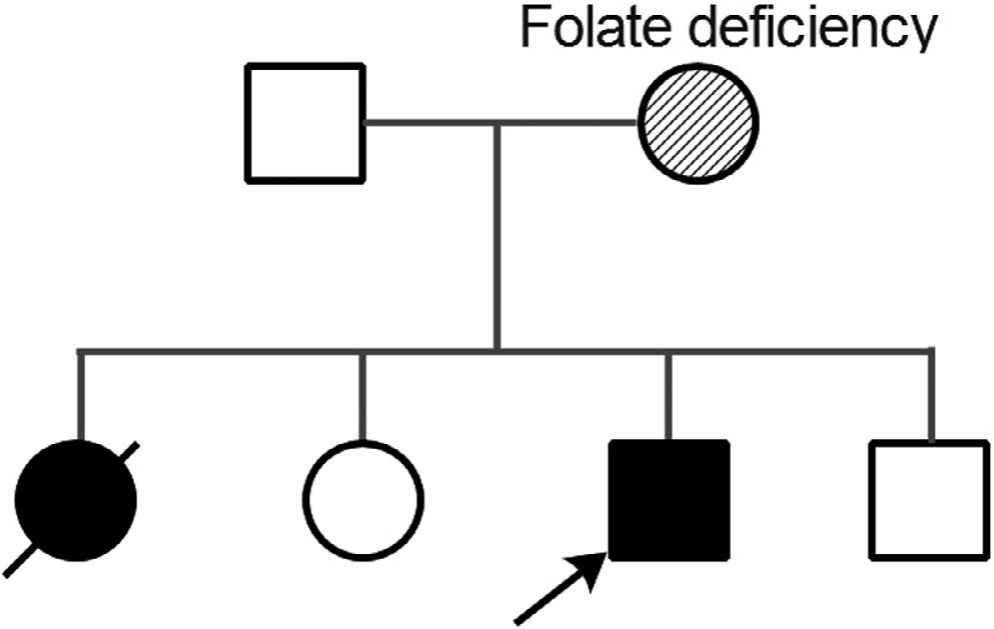
Pedigree of the family. The father of the patient was healthy. The mother complained of folic acid deficiency (transient). The patient had two sisters, one died of a similar disease and the other was healthy. The younger brother had a genetic examination for amniotic fluid before delivery, and he was healthy. Black symbol represents the affected son and daughter by the GFM1 mutations; the arrow represents to the proband; and oblique stripe symbol represents patient with folate deficiency

The patient was the third child, the third delivery, full‐term labor, and spontaneous delivery. There was no history of umbilical cord around the neck and postnatal asphyxia. The birthweight was 2.6 kg and healthy in the neonatal period. At present, the patient cannot sit, stand, or walk. The father of the patient was healthy. The mother complained of folic acid deficiency but no clinical manifestations, and recovered after folic acid supplementation. She supplemented folic acid constantly both before and after pregnancy. The folic acid test of the patient was normal. One of his elder sister (G1P1) had almost the same symptoms as him: developmental retardation, epilepsy, recurrent vomiting, strabismus but not blindness, and so on. She died of seizures at the age of 3. Another elder sister (G2P2) was healthy and did not have the genetic detection. His younger brother had a genetic examination on amniotic fluid before delivery after the patient's gene was confirmed. The results showed that his *GFM1* gene carried heterozygous c.1765‐2‐1765‐1delAG and did not carry c.679G > A (p.G227R) mutation (Figure 1). The study protocol was approved by the ethical committee of Linyi People's Hospital Affiliated to Shandong University (No.13003). The parents signed informed consent forms.

### 2.2 Mutation screening and bioinformatics analysis

Total DNA from peripheral blood leukocytes of the patient and his parents was extracted using the Genomic DNA Extraction Kit (Sangon Biotech) and stored at −20°C prior to use. Gene capture and high‐throughput screening were performed using whole‐exome sequencing (MyGenostics).

Software CASAVA (v1.8.2) was used for base calling of original image data of sequencing results. Short read mapping and alignment with human reference genome (hg19) were performed using BWA software (version 0.7.12, RRID: SCR_010910) (Li & Durbin, 2009). SNV/InDel identification was carried out by using SAMTOOLS software (Li et al., 2009), and the specific sequencing information of each site was analyzed by SAMTOOLS Mpileup module and Bcftools. The detected mutation sites were annotated by ANNOVAR software (RRID: SCR_012821) (Wang, Li, & Hakonarson, 2010). At the same time, we detected the gene package of mitochondrial disease in the patient and his family, and the results were consistent with the sequencing of high‐throughput exons.

## 3 RESULTS

**FIGURE 2:**
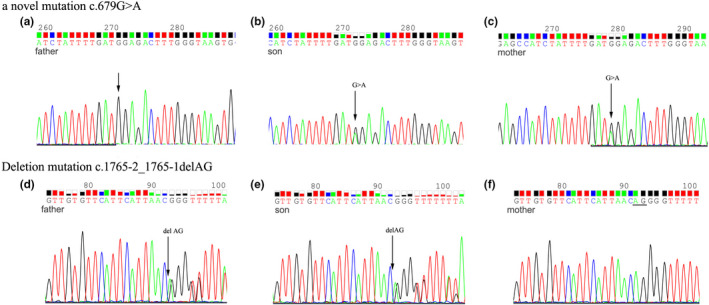
Sequence analysis of the two pathogenic mutations identified in the patient. (a) The wild site c.679G of the father. (b) The novel mutation c.679G > A of the son. (c) The novel mutation c.679G > A of the mother. (d) The deletion mutation C.1765‐2_1765‐1delAG of the father. (e) The deletion mutation C.1765‐2_1765‐1delAG of the son. (f) The wild sites C.1765‐2_1765‐1AG of the mother

**FIGURE 3:**
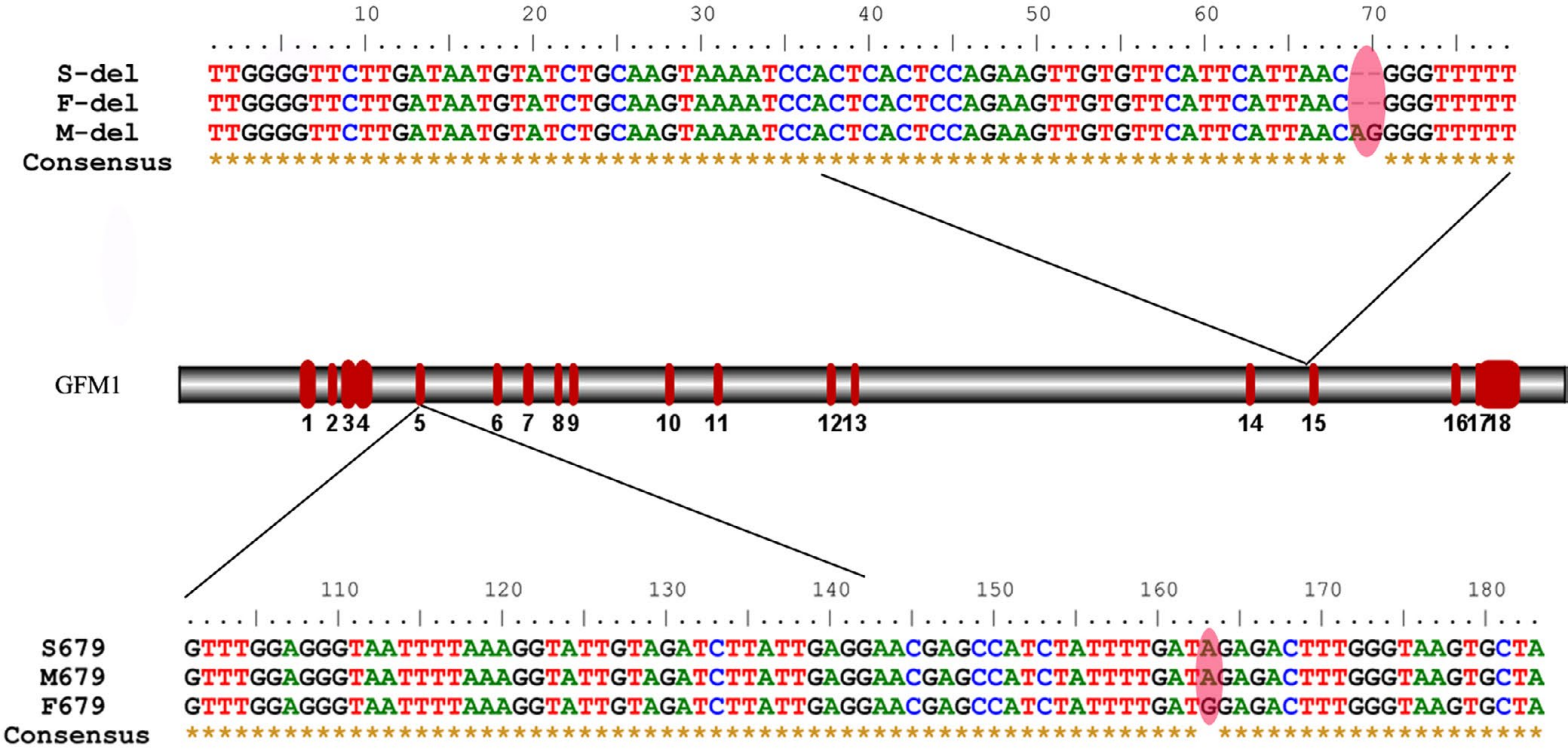
Sequence fragments with the two mutation sites of the family and the locations of the mutations on the gene structure of GFM1

Mutation screening in the affected patient identified a novel composition of two heterozygous mutations of *GFM1* gene, c.679G > A at exon 5 and c.1765‐2_1765‐1delAG deletion at exon 15 (NM_024996). C.679G > A caused a missense mutation from glycine to arginine at position 227 of amino acid sequences. The variation does not belong to polymorphism, and it occurs at a very low frequency in the population. The Human Gene Mutation Database (HGMD) has not reported the mutation. Mutation screening of his parents found a heterozygous mutation in the same site as her mother who was clinically asymptomatic, while no same mutation was found in her father (Figures 2 and 3). C.1765‐2_1765‐1delAG resulted in 2‐bp deletion at the −2 position of exon 15. This variation also does not belong to polymorphism, and it occurs at a very low frequency in the population, and it has been reported to be associated with combined oxidative phosphorylation deficiency in the HGMD. Mutation screening showed that the patient and the father had heterozygous c.1765‐2_1765‐1delAG, whereas the mother had a wild‐type sequence (Figures 2 and 3). The patient's older sister whose symptoms were the same as the patient has died, and her genotype was unknown. His younger brother was healthy and underwent prenatal diagnosis after the patient's gene was confirmed, and he carried the paternal c.1765‐1_1765‐2del but not the maternal c.679G > A.

**FIGURE 4:**
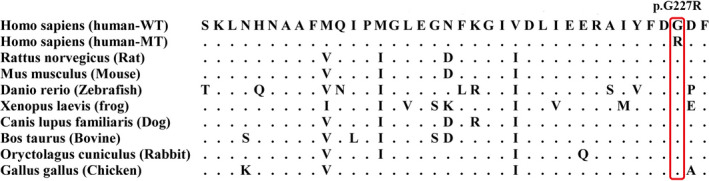
Orthologues of GFM1 from nine available species show a high degree of conservation of the glycine 227 residue

**FIGURE 5:**
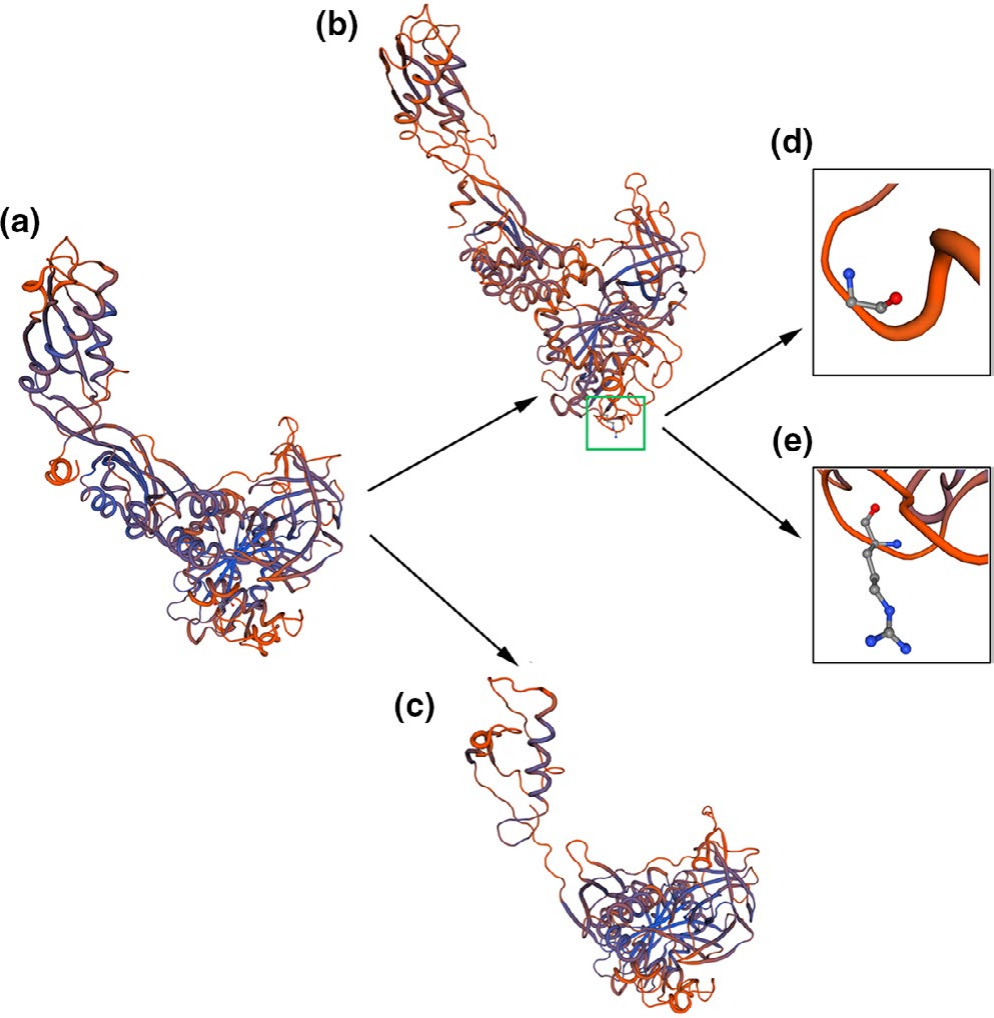
The 3D protein structure of the wild and the variant GFM1. (a) The wild 3D structure of GFM1 protein. (b) The 3D structure of GFM1 protein with c.679G > A of the son. c.679G > A caused a missense mutation from glycine (d) to arginine (e). (c) The 3D structure of GFM1 protein with the deletion mutation C.1765‐2_1765‐1delAG of the son

According to the NCBI RefSeq database, there are fourteen known transcripts of *GFM1* including four noncoding RNAs. The c.679G > A mutation had an effect on transcript variants 1, 2, 3, 4, 6, 8, 9, 11, and 12, while c.1765‐2_1765‐1delAG had an effect on variants 1, 2, 4, 5, 6, 7, 8, 9, 10, 11, and 14. According to the ACMG (American College of Medical Genetics and Genomics) guidelines, we use transcript variant 2 (NM_024996) to analyze the mutations. Transcript variant 2 contains 18 exons and encodes 751 amino acids. C.679G > A resulted in the variation of amino acid 227 of *GFM1* from glycine to arginine. Compared to wide type (WT), the amino sequence in human mutants (MT) is highly conserved in *GFM1* across different species according to multiple sequence alignment in phylogenetic analysis (Figure 4). We synthesized the 3D protein structure of the two variants (Figure 5). As shown in the figure, the 3D structure of GFM1 protein with the deletion mutation C.1765‐2_1765‐1delAG has changed a lot. C.679G > A (Gly227Arg) mutation occurred in the GTP_EFTU domain of EFG1 protein, which is essential for aminoacyl tRNA to enter the A site of the ribosome. Many reported pathogenic *GFM1* mutations were located at this domain. However, there is no study on the specific changes in the protein structure caused by the mutation of this position. Although it is difficult to judge the difference between the structural model of the variant and the wild protein, studies have shown that mutations in this domain do lead to oxidative phosphorylation deficiency (Antonicka, Sasarman, Kennaway, & Shoubridge, 2006; Galmiche et al., 2012).

## 4 DISCUSSION

The prevalence of *GFM1* mutation‐related diseases is unknown. Since the first case of *GFM1*‐linked disease was reported, more than 20 patients have been reported to be associated with *GFM1* gene mutations (Barcia et al., 2020; Coenen et al., 2004). Compound heterozygous mutation is a common type of pathogenic *GFM1* mutation. In a study concerning nine unrelated patients carrying *GFM1* variants (Barcia et al., 2020), eight patients have compound heterozygous *GFM1* mutations. The novel composition of two heterozygous mutations of *GFM1* gene found in our study was c.679G > A and c.1765‐2_1765‐1delAG deletion. C.679G > A (Gly227Arg) was not previously reported. C.1765‐2_1765‐1delAG deletion had been reported in two related patients who also carried another mutation of c.961T > C (Ser321Pro) (Antonicka et al., 2006). The two patients had severe combined oxidative phosphorylation deficiency, one deceased at birth and another suffered from metabolic acidosis, hyperlactatemia, hyperbilirubinemia, and hypoalbuminemia, and deceased at nine days after birth. The clinical features of the two cases were obviously more serious than the case in our study.

**FIGURE 6:**
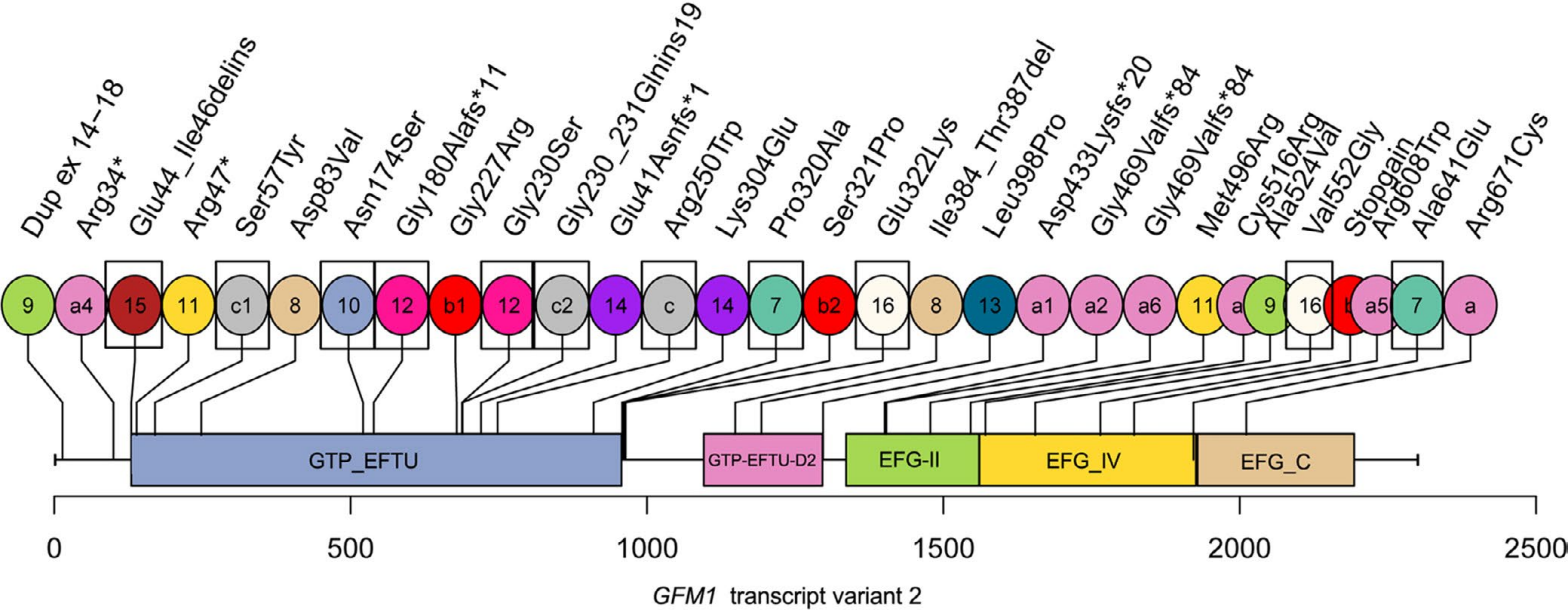
Map of previously reported and novel variant we obtained along the GFM1 protein (transcript variant 2). Circles filled with the same color and the same number represent a set of compound heterozygous mutation. The mutation frequencies of a (c.2011C > T), b (c.1765‐2_1765‐1del), and c (c.748C > T) are higher. They form different complex heterozygous mutations with other mutations (a1‐6, b1, b2, c1, c2). b1(c.679G > A) is the novel mutation we found. Patients with mutations in rectangular boxes had liver involvement

Disease caused by *GFM1* mutations mainly affects the patient's neurologic central nervous system, presented as spasticity, dystonia, epilepsy, and so on. In a study of nine cases, neurological disease and development retardation (including intrauterine growth retardation) were the most common clinical presentations (Barcia et al., 2020). Meanwhile, metabolic workup generally showed elevated lactic acid, as did the case in this study. Some cases also showed elevated cerebrospinal fluid and abnormal pyruvate. Epilepsy was also an important clinical manifestation of *GFM1*‐linked disease, about 41% of patients suffered from epilepsy (Barcia et al., 2020; Calvo et al., 2012; Ravn et al., 2015; Simon et al., 2017; Smits et al., 2011). In this study, seizures occurred at three months old. Although the seizures improved after antiepileptic medication, the patient relapsed and worsened one year later. Liver dysfunction (including hepatomegaly, liver failure, hepatic cytolysis) had attracted much attention in *GFM1*‐related diseases (Antonicka et al., 2006; Balasubramaniam et al., 2012). By mapping some known missense mutations on the crystal structure of the protein, a study suggested that hepatic failure was associated with mutations located in the central part of the protein (Galmiche et al., 2012). However, Barcia et al. showed that the *GFM1* mutations distributed seemingly randomly throughout the mtEFG1 polypeptide, so it is difficult to draw firm genotype–phenotype correlations. According to the statistics of Barcia et al. and the data from ClinVar and MalaCards, we also mapped previously reported and novel variants to the mtEFG1 domains (Figure 6). We found that in the reported cases with liver involvement, the mutations were mainly distributed in the GTP_EFTU domain and its adjacent region, and the EFG‐IV domain. But not all mutations in these two domains were associated with liver dysfunction. Two mutations in the present study were also in the two domains, while the case in this study showed no obvious abnormality of liver function. Other clinical symptoms associated with *GFM*‐linked disease also include microcephaly (Coenen et al., 2004; Simon et al., 2017), feeding difficulties (Smits et al., 2011; Valente et al., 2007), and pyramidal syndrome (Balasubramaniam et al., 2012; Galmiche et al., 2012). The effects of *GFM1* mutations on vision, hearing, kidney, and digestive system have not been reported. Interestingly, the case in our study showed symptoms of blindness and recurrent vomiting, suggesting that *GFM1* mutations may also have an effect on the eyes and digestive system. *GFM1*‐linked disease has various clinical manifestations. It was difficult to determine the genotype–phenotype correlations based on the present cases, but all clinical symptoms mainly focused on nervous system diseases with or without liver involvement, growth retardation, and other aspects. We speculate that the differences in phenotype and severity among different cases with *GFM1*‐linked disease may not be only related to their *GFM1* genotypes, but also related to other genetic factors.

Combined oxidative phosphorylation deficiency was an important reason for the clinical manifestations of *GFM1*‐linked disease (Antonicka et al., 2006; Simon et al., 2017). Diseases caused by *GFM1* mutations were generally classified as oxidative phosphorylation deficiency 1. Many studies suggested that *GFM1* mutation blocked mtDNA encoding 13 protein subunits of respiratory chain complexes, resulting in the decrease in the activity of one or more complexes, thus causing oxidative phosphorylation deficiency (Saada, 2014; Smits, Smeitink, & van den Heuvel, 2010; van Waveren & Moraes, 2008). Metabolic workup had detected mitochondrial oxidative phosphorylation (OXPHOS) complexes deficiency in many *GFM1*‐linked diseases. However, not all tissues detected the complexes deficiency, and not all the complexes had deficiency. For example, Calvo et al. (2012) Calvo et al. detected complex IV deficiency in muscle and fibroblasts in one case, while detecting combined OXPHOS deficiency in liver. Smits et al. (2011) detected OXPHOS multiple deficiency in fibroblasts, but not in muscle. Brito et al. (2015) detected decreased combined OXPHOS deficiency in muscle in their case. Barcia et al. (2020) detected complex I and complex IV deficiency in liver and detected complex IV and complex V deficiency in fibroblasts, but detected normal OXPHOS complex activity in muscle. It is not known what causes the difference. Some researchers believe that EFG1 protein may have tissue‐specific functions, which may be the reason (Coenen et al., 2004). In the present study, the OXPHOS complex activity of the peripheral blood was tested, and the results showed that the activities of the five OXPHOS complexes were all in the normal range. However, this did not mean that OXPHOS complex activity in other tissues was also normal. Besides, the reliability of the measurement of respiratory chain complex activities in blood is not 100%. In a study on the activity of oxidative phosphorylase in peripheral blood leukocytes, 29 of 35 patients with Leigh syndrome (diagnosed with Leigh syndrome based on characteristic brain MRI) were detected with oxidative phosphorylation deficiencies, 20 of which were isolated complex deficiencies (Ma et al., 2011). Based on the clinical phenotype, genotype, and known laboratory data, we believe that there are oxidative phosphorylation defects in other tissues (fibroblasts, muscle, and liver) of the patient.

## 5 CONCLUSIONS

In summary, we reported a novel composition of two heterozygous mutations of *GFM1* gene in a child, c.679G > A and c.1765‐2_1765‐1delAG. Compared with the previously reported *GFM1* mutation related diseases, there are both similarities and differences in the clinical phenotype of this case. The disease caused by *GFM1* gene mutation can involve multiple systems and has a variety of clinical manifestations. At present, there is no specific and effective treatment, and the prognosis is very poor. The relationship between the *GFM1* gene mutation genotype and clinical phenotype, as well as the effective treatment methods, needs to be further studied.

## CONFLICT OF INTEREST

All authors declare that there is no conflict of interest.

## AUTHOR CONTRIBUTION

LY and CPY were responsible for the original concept and the overall design of the research. LY, SYQ, YFL, LYX, and NX analyzed the WES results and diagnosed the patient. XL, CPY, and NX collected the clinical data and sample. LY and CPY carried the experiments and analyzed the sequencing data. LY and CPY wrote and revised the manuscript. All authors read and approved the final manuscript.

### Peer Review

The peer review history for this article is available at https://publons.com/publon/10.1002/brb3.1791.

## Data Availability

The data that support the findings of this study are available on request from the corresponding author. The data are not publicly available due to privacy or ethical restrictions.

## References

[brb31791-bib-0001] Ahola, S. , Isohanni, P. , Euro, L. , Brilhante, V. , Palotie, A. , Pihko, H. , … Suomalainen, A. (2014). Mitochondrial EFTs defects in juvenile‐onset Leigh disease, ataxia, neuropathy, and optic atrophy. Neurology, 83(8), 743–751. 10.1212/WNL.0000000000000716 25037205 PMC4150129

[brb31791-bib-0002] Antonicka, H. , Sasarman, F. , Kennaway, N. G. , & Shoubridge, E. A. (2006). The molecular basis for tissue specificity of the oxidative phosphorylation deficiencies in patients with mutations in the mitochondrial translation factor EFG1. Human Molecular Genetics, 15(11), 1835–1846. 10.1093/hmg/ddl106 16632485

[brb31791-bib-0003] Balasubramaniam, S. , Choy, Y. , Talib, A. , Norsiah, M. , van den Heuvel, L. , & Rodenburg, R. (2012). Infantile progressive hepatoencephalomyopathy with combined OXPHOS deficiency due to mutations in the mitochondrial translation elongation factor gene GFM1. JIMD Reports, 5, 113–122. 10.1007/8904_2011_107 23430926 PMC3509912

[brb31791-bib-0004] Barcia, G. , Rio, M. , Assouline, Z. , Zangarelli, C. , Gueguen, N. , Dumas, V. D. , … Barth, M. (2020). Clinical, neuroimaging and biochemical findings in patients and patient fibroblasts expressing ten novel GFM1 mutations. Human Mutation, 41(2), 397–402. 10.1002/humu.23937 31680380

[brb31791-bib-0005] Brito, S. , Thompson, K. , Campistol, J. , Colomer, J. , Hardy, S. A. , He, L. , … Taylor, R. W. (2015). Long‐term survival in a child with severe encephalopathy, multiple respiratory chain deficiency and GFM1 mutations. Frontiers in Genetics, 6, 102 10.3389/fgene.2015.00102 25852744 PMC4369643

[brb31791-bib-0006] Calvo, S. E. , Compton, A. G. , Hershman, S. G. , Lim, S. C. , Lieber, D. S. , Tucker, E. J. , … Mootha, V. K. (2012). Molecular diagnosis of infantile mitochondrial disease with targeted next‐generation sequencing. Science Translational Medicine, 4(118), 118ra110 10.1126/scitranslmed.3003310 PMC3523805 22277967

[brb31791-bib-0007] Coenen, M. J. H. , Antonicka, H. , Ugalde, C. , Sasarman, F. , Rossi, R. , Heister, J. G. A. M. A. , … Smeitink, J. A. M. (2004). Mutant mitochondrial elongation factor G1 and combined oxidative phosphorylation deficiency. New England Journal of Medicine, 351(20), 2080–2086. 10.1056/NEJMoa041878 15537906

[brb31791-bib-0008] de Laat, P. , Rodenburg, R. , & Smeitink, J. (2014). Mitochondrial oxidative phosphorylation disorders In Blau N., Duran M., Blaskovics M. E., & Gibson K. M. (Eds.), Physician's guide to the diagnosis, treatment, and follow‐up of inherited metabolic diseases (pp. 337–359). Berlin, Germany: Springer.

[brb31791-bib-0009] Fukumura, S. , Ohba, C. , Watanabe, T. , Minagawa, K. , Shimura, M. , Murayama, K. , … Tsutsumi, H. (2015). Compound heterozygous GFM2 mutations with Leigh syndrome complicated by arthrogryposis multiplex congenita. Journal of Human Genetics, 60(9), 509–513. 10.1038/jhg.2015.57 26016410

[brb31791-bib-0010] Galmiche, L. , Serre, V. , Beinat, M. , Zossou, R. , Assouline, Z. , Lebre, A.‐S. , … Rötig, A. (2012). Toward genotype phenotype correlations in GFM1 mutations. Mitochondrion, 12(2), 242–247. 10.1016/j.mito.2011.09.007 21986555

[brb31791-bib-0011] Gao, J. , Yu, L. , Zhang, P. , Jiang, J. , Chen, J. , Peng, J. , … Zhao, S. (2001). Cloning and characterization of human and mouse mitochondrial elongation factor G, GFM and Gfm, and mapping of GFM to human chromosome 3q25.1–q26.2. Genomics, 74(1), 109–114. 10.1006/geno.2001.6536 11374907

[brb31791-bib-0012] Glasgow, R. I. C. , Thompson, K. , Barbosa, I. A. , He, L. , Alston, C. L. , Deshpande, C. , … Taylor, R. W. (2017). Novel GFM2 variants associated with early‐onset neurological presentations of mitochondrial disease and impaired expression of OXPHOS subunits. Neurogenetics, 18(4), 227–235. 10.1007/s10048-017-0526-4 29075935 PMC5705740

[brb31791-bib-0013] Hershkovitz, T. , Kurolap, A. , Gonzaga‐Jauregui, C. , Paperna, T. , Mory, A. , Wolf, S. E. , … Baris Feldman, H. (2019). A novel TUFM homozygous variant in a child with mitochondrial cardiomyopathy expands the phenotype of combined oxidative phosphorylation deficiency 4. Journal of Human Genetics, 64(6), 589–595. 10.1038/s10038-019-0592-6 30903008

[brb31791-bib-0014] Kuzmenko, A. , Atkinson, G. C. , Levitskii, S. , Zenkin, N. , Tenson, T. , Hauryliuk, V. , & Kamenski, P. (2014). Mitochondrial translation initiation machinery: Conservation and diversification. Biochimie, 100, 132–140. 10.1016/j.biochi.2013.07.024 23954798 PMC3978653

[brb31791-bib-0015] Li, H. , & Durbin, R. (2009). Fast and accurate short read alignment with Burrows‐Wheeler transform. Bioinformatics, 25(14), 1754–1760. 10.1093/bioinformatics/btp324 19451168 PMC2705234

[brb31791-bib-0016] Li, H. , Handsaker, B. , Wysoker, A. , Fennell, T. , Ruan, J. , Homer, N. , … Durbin, R. (2009). The sequence alignment/map format and SAMtools. Bioinformatics, 25(16), 2078–2079. 10.1093/bioinformatics/btp352 19505943 PMC2723002

[brb31791-bib-0017] Ma, Y.‐Y. , Zhang, X.‐L. , Wu, T.‐F. , Liu, Y.‐P. , Wang, Q. , Zhang, Y. , … Yang, Y.‐L. (2011). Analysis of the mitochondrial complex I‐V enzyme activities of peripheral leukocytes in oxidative phosphorylation disorders. Journal of Child Neurology, 26(8), 974–979. 10.1177/0883073811399905 21540367

[brb31791-bib-0018] Ravn, K. , Schönewolf‐Greulich, B. , Hansen, R. M. , Bohr, A.‐H. , Duno, M. , Wibrand, F. , & Ostergaard, E. (2015). Neonatal mitochondrial hepatoencephalopathy caused by novel GFM1 mutations. Molecular Genetics and Metabolism Reports, 3, 5–10. 10.1016/j.ymgmr.2015.01.004 26937387 PMC4750589

[brb31791-bib-0019] Saada, A. (2014). Mitochondria: Mitochondrial OXPHOS (dys) function ex vivo–the use of primary fibroblasts. International Journal of Biochemistry & Cell Biology, 48, 60–65. 10.1016/j.biocel.2013.12.010 24412346

[brb31791-bib-0020] Scala, M. , Brigati, G. , Fiorillo, C. , Nesti, C. , Rubegni, A. , Pedemonte, M. , … Santorelli, F. M. (2019). Novel homozygous TSFM pathogenic variant associated with encephalocardiomyopathy with sensorineural hearing loss and peculiar neuroradiologic findings. Neurogenetics, 20(3), 165–172. 10.1007/s10048-019-00582-5 31267352

[brb31791-bib-0021] Scheffer, I. E. , Berkovic, S. , Capovilla, G. , Connolly, M. B. , French, J. , Guilhoto, L. , … Zuberi, S. M. (2017). ILAE classification of the epilepsies: Position paper of the ILAE Commission for Classification and Terminology. Epilepsia, 58(4), 512–521. 10.1111/epi.13709 28276062 PMC5386840

[brb31791-bib-0022] Simon, M. T. , Ng, B. G. , Friederich, M. W. , Wang, R. Y. , Boyer, M. , Kircher, M. , … Abdenur, J. E. (2017). Activation of a cryptic splice site in the mitochondrial elongation factor GFM1 causes combined OXPHOS deficiency. Mitochondrion, 34, 84–90. 10.1016/j.mito.2017.02.004 28216230 PMC5444868

[brb31791-bib-0023] Smits, P. , Antonicka, H. , van Hasselt, P. M. , Weraarpachai, W. , Haller, W. , Schreurs, M. , … van den Heuvel, L. P. (2011). Mutation in subdomain G'of mitochondrial elongation factor G1 is associated with combined OXPHOS deficiency in fibroblasts but not in muscle. European Journal of Human Genetics, 19(3), 275–279. 10.1038/ejhg.2010.208 21119709 PMC3062000

[brb31791-bib-0024] Smits, P. , Smeitink, J. , & van den Heuvel, L. (2010). Mitochondrial translation and beyond: processes implicated in combined oxidative phosphorylation deficiencies. Journal of Biomedicine and Biotechnology, 2010, 1–24. 10.1155/2010/737385 PMC2854570 20396601

[brb31791-bib-0025] Thompson, K. , Collier, J. J. , Glasgow, R. I. C. , Robertson, F. M. , Pyle, A. , Blakely, E. L. , … Taylor, R. W. (2020). Recent advances in understanding the molecular genetic basis of mitochondrial disease. Journal of Inherited Metabolic Disease, 43(1), 36–50. 10.1002/jimd.12104 31021000 PMC7041634

[brb31791-bib-0026] Valente, L. , Tiranti, V. , Marsano, R. M. , Malfatti, E. , Fernandez‐Vizarra, E. , Donnini, C. , … Zeviani, M. (2007). Infantile encephalopathy and defective mitochondrial DNA translation in patients with mutations of mitochondrial elongation factors EFG1 and EFTu. American Journal of Human Genetics, 80(1), 44–58. 10.1086/510559 17160893 PMC1785320

[brb31791-bib-0027] van Waveren, C. , & Moraes, C. T. (2008). Transcriptional co‐expression and co‐regulation of genes coding for components of the oxidative phosphorylation system. BMC Genomics, 9(1), 18 10.1186/1471-2164-9-18 18194548 PMC2268925

[brb31791-bib-0028] Wang, K. , Li, M. , & Hakonarson, H. (2010). ANNOVAR: Functional annotation of genetic variants from high‐throughput sequencing data. Nucleic Acids Research, 38(16), e164 10.1093/nar/gkq603 20601685 PMC2938201

